# ChatGPT for Future Medical and Dental Research

**DOI:** 10.7759/cureus.37285

**Published:** 2023-04-08

**Authors:** Bader Fatani

**Affiliations:** 1 Dentistry, College of Dentistry, King Saud University, Riyadh, SAU

**Keywords:** research, dentistry, medicine, chatgpt, artificial intelligence

## Abstract

ChatGPT is an artificial intelligence (AI) chatbot developed by OpenAI and it first became available to the public in November 2022. ChatGPT can assist in finding academic papers on the web and summarizing them. This chatbot has the potential to be applied in scientific writing, it has the ability to generate automated drafts, summarize articles, and translate content from several languages. This in turn can make academic writing faster and less challenging. However, due to ethical considerations, its use in scientific writing should be regulated and carefully monitored. Few papers have discussed the use of ChatGPT in scientific research writing. This review aims to discuss all the relevant published papers that discuss the use of ChatGPT in medical and dental research.

## Introduction and background

ChatGPT is an artificial intelligence (AI) program that generates text based on written prompts; this program has gained a great deal of popularity as evidenced by its web-based accessibility through OpenAI [1]. ChatGPT is a natural language processing model with 175 billion parameters that uses deep learning algorithms to generate responses similar to those of humans. As a versatile conversational agent, it can handle various topics, making it useful for customer service, chatbots, and other applications. Though it has received a lot of attention for exceptional features such as generating Shakespearean sonnets, its inability to answer straightforward math questions has also been noted [2].

ChatGPT was trained through reinforcement learning using human feedback, using the same methods as InstructGPT (a generative pre-trained transformer model). Following its public release on November 30, 2022, ChatGPT has gained a lot of attention, especially in the field of education [3]. ChatGPT is an improved version of OpenAI's GPT-3.5 language models developed by incorporating reinforcement and supervised learning methods. It is a refined descendant of InstructGPT, a variant of GPT-3.5 that was fine-tuned on human-generated responses to prompts submitted to the OpenAI API Playground. InstructGPT was trained using a reinforcement learning algorithm called Proximal Policy Optimization, which maximized the preferences of human annotators for specific prompts. ChatGPT, on the other hand, has been specifically designed and trained with conversational prompts to encourage dialogic output [2].

Language models have been explored as tools for consumer health education and personalized patient interaction in the medical domain. While showing promise, these models have not succeeded in testing clinical knowledge. ChatGPT offers a new generation of models that could better combine clinical knowledge and dialogic interaction. Its unique narrative interface allows for innovative applications, such as simulating as a patient, a brainstorming tool, or a fellow student for small-group-style learning. However, for these efforts to be successful, ChatGPT must perform similarly to humans in evaluating medical knowledge and reasoning so that users can rely on its responses with confidence [2]. The Huh study aimed to compare the interpretation and knowledgeability of ChatGPT compared to medical students in Korea. The study explained that ChatGPT's capability to understand and interpret parasitology exam questions is still not on par with Korean medical students [3]. On the other hand, Gilson et al. aimed to investigate and observe ChatGPT's performance in the United States Medical Licensing Examination. The authors reported that the model achieved a passing score for a medical student in the third year by performing above 60% on the NBME-Free-Step-1 dataset [2]. Sabry et al. analyzed ChatGPT using a case of clinical toxicology of acute organophosphate poisoning. As reported by the authors, ChatGPT was able to answer all of the questions regarding the introduced case [4]. The architecture of ChatGPT is derived from Generative Pre-trained Transformer 3 (GPT-3 x) and it has been trained on a vast amount of data. It is being integrated into the Microsoft Bing search engine, which will make it easily accessible to a large number of users worldwide, including patients, nursing and medical students, and clinicians [4].

Program directors should familiarize themselves with the overall characteristics of this technology and take into account its impact on graduate medical education, including the creation of documents like personal statements [5]. The study by Rao et al. evaluated the capacity of ChatGPTs in terms of providing help in clinical decision-making in radiology by identification of appropriate imaging services for breast pain and breast cancer screening clinical presentations [6]. According to the authors, using ChatGPT for radiologic decision-making has been shown to be possible and has the potential to enhance clinical workflow while promoting the responsible use of radiology services [6].

## Review

Methods

This narrative review involved an evaluation of published papers discussing the use of ChatGPT for medical and dental research. The population, intervention, control, and outcomes (PICO) framework was used to address the following question: in medical and dental research, is ChatGPT effective in writing scientific papers?

Databases such as PubMed and Google Scholar were used to gather the most relevant papers. A search set included a range of keywords such as ChatGPT and research. By using this method, all the papers discussing the use of ChatGPT for medical and dental research were obtained. We included all the relevant papers discussing ChatGPT and research in the inclusion criteria. Studies that discussed the use of ChatGPT in medical and dental research were deemed eligible. The studies that were out of scope were excluded. The initial screening elicited 69 papers. The most appropriate papers were chosen based on our inclusion criteria and used in the current narrative review. Ultimately, we reviewed 20 papers related to ChatGPT for medical and dental research.

ChatGPT in medical and dental research

AI-powered language models are becoming increasingly popular in data analysis and scientific writing [7]. The AI language model ChatGPT can assist medical researchers and scientists in writing, literature research, summarizing data, suggesting structures, references, and titles, and even generating an initial paper draft. However, this is just a beginning point for humans to develop the text further. ChatGPT can assist in finding academic papers, summarizing their conclusions, and highlighting areas of uncertainty, but the provided summary might lack critical analysis of differences among studies. AI can potentially help in generating figures, tables, and other visual elements to summarize data. ChatGPT's main advantage is its ability to understand information quickly and connect evidence to reach conclusions faster than humans who have limitations in reading a wide range of literature comprehensively and connecting seemingly disparate pieces of information [8]. Basically, anyone can ask for anything in a conversational way and get a quick and satisfactory response that resembles human writing. This can include tasks such as writing a short text on a specific subject, obtaining information on a topic of interest, composing an email or message with specific content, tone, and intended recipient, modifying the format or wording of a particular text, and solving problems [8]. This chatbot has the potential to be applied in scientific writing. With its ability to generate automated drafts, summarize articles, and translate languages, it can make academic writing faster and less challenging.

Nonetheless, due to ethical concerns, its use in scientific writing should be regulated [8]. Nowadays, writing assistance tools, especially in English, offer more than just checks for grammar, punctuation, and spelling errors. They can quickly suggest synonyms for words and even modify the tone and style of the text by paraphrasing. With the use of AI-powered content generation tools like ChatGPT, authors can generate multiple versions of their text in just a few seconds, which could potentially help them overcome writer's block [9]. AI technology has the potential to assist with basic research and transform clinical and translational medicine. It can use image recognition to identify and describe chemical formulas and molecular structures to help design new compounds. Additionally, AI helps predict and diagnose diseases and provide treatment guidance for patients based on their imaging and other factors [10]. ChatGPT can serve as a useful tool for building upon an existing text, improving material, and rewording content as necessary. However, as medical research is constantly evolving, there is a growing worry that ChatGPT may be misused to produce papers lacking in clinical reasoning and critical thinking [11]. Certain scientific journals now require that the researchers who produce articles with ChatGPT-generated content list ChatGPT as an author. In contrast, Nature has declined to accept ChatGPT as an author because it cannot take responsibility for content created by ChatGPT itself.

AI models such as ChatGPT can contribute to the healthcare sector by offering an objective and evidence-backed method of decision-making, thereby decreasing the likelihood of human error due to its unmatched processing speed. Furthermore, it has the potential to uncover new insights and discoveries in medicine by identifying patterns and correlations in massive quantities of data [10].

AI's utilization in academia has been extensively deliberated in the scientific community, and concerns are being raised about the efficacy of these technological tools in producing high-quality research papers and influencing research areas. The primary concern involves plagiarism. Little is currently known about this developing field [12]. AI is widely used in biotechnology for various purposes, ranging from drug discovery and safety to genomics, proteomics, metabolomics, pharmacology, pharmacogenetics, and pharmacogenomics [13]. ChatGPT could make a significant impact on clinical and translational medicine fields by enhancing patient involvement, reducing healthcare provider workloads, and providing up-to-date information. However, to guarantee the secure and efficient application of ChatGPT, there are obstacles and issues that need to be evaluated and addressed through ongoing research and development [14]. Ali et al. have examined using ChatGPT to write patient clinical letters [15]. According to a pilot assessment, ChatGPT can generate clinic letters with high scores for correctness, at a reading level similar to human-written letters. However, the use of AI in healthcare communication must be regulated and monitored to avoid potential risks, such as errors or misinterpretations that could harm patients. To responsibly incorporate ChatGPT, a possible method could be to use voice-to-text identification software with rapid clinician editing of the letter and limited human input. This would allow for exploring the potential applications of the technology while mitigating any potential risks [15].

The increasing focus on improving treatment efficacy in dental practice has resulted in the creation of various tools, including Dental Monitoring (DM) software and White Teeth, which incorporates AI and telemedicine. DM allows for effortless daily cooperation and interaction between dental practices and patients via a smartphone software app, allowing for the coordination of each treatment phase and the monitoring of treatment outcomes. Both parties can use the tool to its full potential [16]. The study by Strunga et al. investigated the use of AI software in orthodontics [16]. The authors concluded that AI is a promising field for improving patient care and results in orthodontic treatment, and we can expect to see more AI-powered tools and systems developed and adopted in this area. However, current literature shows that these systems should be used alongside trained orthodontists in order to achieve the best outcomes, and unsupervised use of AI-assisted orthodontic treatment is inconsistent with medical ethics and standards of good practice. This is due in part to the risks of AI bias, as well as new regulatory considerations for AI in healthcare that are on the horizon [16].

Recommendations for the future

ChatGPT has become the AI consumer application with the highest rate of growth up until this point, surpassing 100 million users in January 2023 [17]. Although ChatGPT is able to generate credible scientific essays, the fact that the data it provided includes both truthful and completely fabricated information has caused concern about its accuracy and integrity in academic writing. Policies and practices for evaluating scientific manuscripts should be adopted to ensure strict scientific standards. Implementation of AI output detectors in the editorial process and a clear declaration of their use should also be considered. The ethical and acceptable use of large language models in scientific writing is still a matter of debate, as they could potentially create false experts in the medical field and cause harm due to the lack of real experience and the generation of dubious "expert" opinions through ChatGPT [18].

It is important to take into account the possible difficulties and drawbacks of using ChatGPT, including ethical concerns and negative impacts. Medical educators must stay up-to-date with the rapid advancements in technology and consider how it affects their teaching approaches, curriculum development, and evaluation methods [19]. Using AI to administer standardized tests could perpetuate biases found in the data used to train these models. Furthermore, there have been instances of flawed and biased proprietary algorithms being implemented without proper evaluation. It is important to note that AI can never fully replace the essential roles of healthcare professionals such as nurses and doctors [20]. Writing is crucial for research, and while ChatGPT should not be the primary source of content, it can greatly assist with improving language and spotting errors. This is particularly beneficial for people who are not native speakers of the used language. ChatGPT can also be utilized to translate text with personalized requests and summarize critical information from lengthy material. Despite constant updates and enhancements made to ChatGPT, eliminating false or fake information completely is extremely difficult. Additionally, relying solely on AI to solve environmental problems is controversial due to the social bias that ChatGPT is associated with, and the fact that AI cannot yet be held accountable for its decisions. Therefore, when AI is involved in decision-making processes related to public welfare, extreme caution must be exercised [21].

To ensure the proper use of ChatGPT and avoid any unexpected issues, researchers and practitioners must have a good understanding of its capabilities and limitations. By recognizing these limitations, they can also determine areas where the model needs improvement. However, due to significant constraints, using these tools for clinical aid and research presents several difficulties [22]. ChatGPT has various applications apart from research, such as providing consultation to patients, support to patients, and marketing [23]. Researchers can assign monotonous work like manuscript editing to ChatGPT by being aware of its limitations, thereby preventing errors like false information from being published. It is critical to set reasonable expectations for the growing use of ChatGPT and recognize its inability to perform every task. In the academic field, specific tasks requiring expertise or innovative ideas necessitate human intervention that AI cannot replace [24]. It is crucial to encourage the responsible use of ChatGPT among potential users and to ensure proper citation of any sources referenced. There are concerns about the ability of current plagiarism checkers in journal Editorial Managers to detect instances of plagiarism resulting from the use of ChatGPT. These tools may not be adequate or sensitive enough to identify such instances of plagiarism caused by chatbots [25]. New and innovative methods for detecting AI usage are required and need to be introduced [26]. Moreover, just because AI cannot be a copyright holder does not mean it cannot be registered as an author. If the writing was not done by a human, it might not make sense to attribute it to a human author [27]. As technology advances, it is getting harder to tell if written content is truly original or if it was created by a machine. This poses questions about the importance of originality and proper source attribution in the digital age. It also emphasizes the need for people to be more discerning about the information they consume and share, as well as the fact that the rapid development of AI and chatbot technology is forcing us to rethink our understanding of attribution and originality in the digital realm [28].

## Conclusions

ChatGPT has proven its ability in assisting researchers with medical and dental research writing, as reported by previously published papers. The use of ChatGPT has enabled researchers to summarize, translate, and paraphrase scientific information. However, being fully dependent on ChatGPT for research writing is not advisable since scientific writing generated by this chatbot has not yet been fully evaluated and more studies are required to investigate the ethical concerns and negative impacts of this program.

## References

[1] Thorp HH (2023). ChatGPT is fun, but not an author. Science.

[2] Gilson A, Safranek CW, Huang T, Socrates V, Chi L, Taylor RA, Chartash D (2023). How does ChatGPT perform on the United States Medical Licensing Examination? The implications of large language models for medical education and knowledge assessment. JMIR Med Educ.

[3] Huh S (2023). Are ChatGPT’s knowledge and interpretation ability comparable to those of medical students in Korea for taking a parasitology examination?: a descriptive study. J Educ Eval Health Prof.

[4] Sabry Abdel-Messih M, Kamel Boulos MN (2023). ChatGPT in clinical toxicology. JMIR Med Educ.

[5] Zumsteg JM, Junn C (2023). Will ChatGPT match to your program?. Am J Phys Med Rehabil.

[6] Rao A, Kim J, Kamineni M, Pang M, Lie W, Succi MD (2023). Evaluating ChatGPT as an adjunct for radiologic decision-making. medRxiv.

[7] Macdonald C, Adeloye D, Sheikh A, Rudan I (2023). Can ChatGPT draft a research article? An example of population-level vaccine effectiveness analysis. J Glob Health.

[8] Salvagno M, Taccone FS, Gerli AG (2023). Can artificial intelligence help for scientific writing?. Crit Care.

[9] Chen TJ (2023). ChatGPT and other artificial intelligence applications speed up scientific writing. J Chin Med Assoc.

[10] Xue VW, Lei P, Cho WC (2023). The potential impact of ChatGPT in clinical and translational medicine. Clin Transl Med.

[11] Arif TB, Munaf U, Ul-Haque I (2023). The future of medical education and research: Is ChatGPT a blessing or blight in disguise?. Med Educ Online.

[12] Jungwirth D, Haluza D (2023). Artificial intelligence and public health: an exploratory study. Int J Environ Res Public Health.

[13] Holzinger A, Keiblinger K, Holub P, Zatloukal K, Müller H (2023). AI for life: Trends in artificial intelligence for biotechnology. N Biotechnol.

[14] Baumgartner C (2023). The potential impact of ChatGPT in clinical and translational medicine. Clin Transl Med.

[15] Ali SR, Dobbs TD, Hutchings HA, Whitaker IS (2023). Using ChatGPT to write patient clinic letters. Lancet Digit Health.

[16] Strunga M, Urban R, Surovková J, Thurzo A (2023). Artificial intelligence systems assisting in the assessment of the course and retention of orthodontic treatment. Healthcare (Basel).

[17] Eysenbach G (2023). The role of ChatGPT, generative language models, and artificial intelligence in medical education: a conversation with ChatGPT and a call for papers. JMIR Med Educ.

[18] Alkaissi H, McFarlane SI (2023). Artificial hallucinations in ChatGPT: implications in scientific writing. Cureus.

[19] Lee H (2023). The rise of ChatGPT: exploring its potential in medical education. Anat Sci Educ.

[20] Mbakwe AB, Lourentzou I, Celi LA, Mechanic OJ, Dagan A (2023). ChatGPT passing USMLE shines a spotlight on the flaws of medical education. PLOS Digit Health.

[21] Zhu JJ, Jiang J, Yang M, Ren ZJ (2023). ChatGPT and environmental research. Environ Sci Technol.

[22] Cascella M, Montomoli J, Bellini V, Bignami E (2023). Evaluating the feasibility of ChatGPT in healthcare: an analysis of multiple clinical and research scenarios. J Med Syst.

[23] Gupta R, Park JB, Bisht C (2023). Expanding cosmetic plastic surgery research using ChatGPT. Aesthet Surg J.

[24] Wen J, Wang W (2023). The future of ChatGPT in academic research and publishing: A commentary for clinical and translational medicine. Clin Transl Med.

[25] Dahmen J, Kayaalp ME, Ollivier M, Pareek A, Hirschmann MT, Karlsson J, Winkler PW (2023). Artificial intelligence bot ChatGPT in medical research: the potential game changer as a double-edged sword. Knee Surg Sports Traumatol Arthrosc.

[26] Anderson N, Belavy DL, Perle SM, Hendricks S, Hespanhol L, Verhagen E, Memon AR (2023). AI did not write this manuscript, or did it? Can we trick the AI text detector into generated texts? The potential future of ChatGPT and AI in sports & exercise medicine manuscript generation. BMJ Open Sport Exerc Med.

[27] Lee JY (2023). Can an artificial intelligence chatbot be the author of a scholarly article?. J Educ Eval Health Prof.

[28] King MR (2023). A conversation on artificial intelligence, chatbots, and plagiarism in higher education. Cell Mol Bioeng.

